# Using AI to prepare for academic interviews – don’t trade authenticity for polish

**DOI:** 10.1038/s44319-025-00396-7

**Published:** 2025-02-17

**Authors:** Shina Caroline Lynn Kamerlin, William C Ratcliff

**Affiliations:** 1 Georgia Institute of Technology School of Chemistry and Biochemistry USA, Atlanta, GA USA; 2 Georgia Institute of Technology School of Biological Sciences USA, Atlanta, GA USA

**Keywords:** Careers

## Abstract

An increasingly popular application of AI is to prepare for job interviews. However, this is one use that may actually harm rather than benefit applicants.

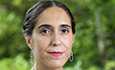

The widespread adoption of large language models (LLMs) such as ChatGPT has fundamentally changed how candidates approach academic recruitment, including job interviews. While these tools offer obvious benefits for preparing an application, particularly for non-native English speakers, their use raises important questions about how we evaluate and select candidates for academic positions. Our experience on faculty search, postdoc hiring and graduate admission committees suggests that overreliance on AI-assisted preparation may actually harm, rather than help, candidates’ chances of success.

Since its release in November 2022, ChatGPT and other large language models (LLMs) have propelled generative artificial intelligence into the mainstream. While there are many benefits to LLMs—for instance, in our fields, it can be very helpful for specific coding and writing workflows—not all applications are necessarily helpful for the user. Here, we will focus on a new phenomenon both authors of this column have been observing: the use of generative AI for preparing application packages and job interviews. Their uptake to help with writing cover letters and personal statements has been rapid. More recently, we are also observing the use of generative AI to prepare for interviews, for instance, for graduate school, postdocs or faculty positions, all areas where we are involved in recruitment. While we do not see anything inherently problematic with candidates using LLMs to help organize their thoughts, our experience suggests that it may do more harm than good during the application process.

From a practical perspective, the benefits may seem obvious, especially for non-native English speakers, as AI-based tools can help refine text, as well as practice prospective interview questions. In fact, there are countless online websites advocating the use of AI for preparing for an interview while providing tips, tools and suggestions on how to do so (see, for instance, Google’s Interview Warmup, advice from the Oregon State Alumni Association and even Harvard University, among others). Clearly, in a hyper-competitive environment, preparing a convincing application package and subsequent interview for a position one is deeply invested in is, as an understatement, extremely stressful. This is in particular amplified for non-native English speakers who are not operating in their primary language. The use of generative AI for improving linguistic structure can come across as a helpful solution to this problem.

However, there is a reason a personal statement contains the word *personal*. In a competitive selection process, a key part of what makes an application and applicant stand out are the unique characteristics and experiences they bring to their portfolio. Content is more important than language. What a selection committee wants to see is who *you* are. Why are you passionate about the post? Why do you want to pursue a PhD, work as a postdoc or join a department as faculty? What experience and background will you bring with you? LLMs, by design, generate outputs that regress towards the mean of their training data, thereby removing your greatest strength as an applicant: you. Thus, while there are areas where the use of an LLM can polish your application, it can also hide key strengths that you bring to the table that make you unique and stand out among the pool of candidates. It also makes it difficult to the recruiter or the hiring committee to assess the benefit of recruiting you specifically rather than other applicants in the pool.

This is already an issue with written work, but amplified even further in oral interviews, where we have observed candidates who appear to have over-prepared by memorizing answers generated by an LLM instead of showing off the best side of themselves. An interviewee who merely parrots AI-generated responses can come across as artificial and wooden. For a strong application package, we therefore recommend the following advice. First, make your personal statement personal. Read the position description carefully and describe why you are a good match for the position. Bring out what makes you *you*.

Second, if you are invited to an interview, be familiar with both the position you are applying for, including the department and program you would be part of, and your own application materials. What interviewers are probing at this stage is what makes you unique and what makes you a good match for the position. This is not something you can really prepare for. If it helps, write down prospective questions—you can easily find “common interview questions” for the specific level of position you are applying for on the internet—and prepare mock answers, but do not over-practice these. Be prepared to answer entirely different questions on the actual interview. One approach that may be helpful is the use of LLMs to act as mock interviewers—for instance, using ChatGPT in voice mode—which provides an opportunity to practice answering standard interview questions and follow-ups.

Lastly, while these tools can be valuable for preparing an application, it is crucial to avoid them during the actual interview, especially in virtual settings where real-time AI assistance might seem tempting. If your interview is a relaxed discussion, where both you and the interviewers enjoy talking to each other about science or any other topic, you are most likely to have a positive outcome. Prepare, but do not over-prepare, as over-preparation tends to undermine the more natural discussion style that is a hallmark of a great interview.

Overall, it’s clear that generative AI is here to stay. There are situations where it can be beneficial and helpful, and if, as a tool, improves one’s writing, science or computer code, then this provides a net good for science and society. However, it’s crucial for AI to be used responsibly. In the context of an interview, we think applicants generally have more to lose than to gain with an overreliance on AI. Anthropic, the creator of the popular LLM Claude, appears to agree with us: the company now explicitly requires job applicants to confirm they will not use AI assistants during their application process. As a rule of thumb, do not let an AI generate content for you—no matter how good AI gets at mirroring human thought and speech, there is still nothing better than you.

## Supplementary information

Peer Review File

