# Supplementary material for: Using AI to prepare for academic interviews – don’t trade authenticity for polish
Source: EMBO Rep. 2025 Feb 17;26(5):1189–90. doi: 10.1038/s44319-025-00396-7 (PMC11894150; doi:10.1038/s44319-025-00396-7)
Supplement: Supplementary file 1 — Peer Review File [file 44319_2025_396_MOESM1_ESM.pdf]

EMBO reports

Using AI to Prepare for Academic Interviews - Don't Trade Authenticity for Polish

William Ratcliff and Shina Caroline Lynn Kamerlin

Corresponding author(s): Shina Caroline Lynn Kamerlin (skamerlin3@gatech.edu)

Review Timeline:	Submission Date:	4th Feb 25
	Accepted:	6th Feb 25

Editor: Holger Breithaupt

Transaction Report: This article type is not peer reviewed.
